# Supplementary material for: Stable and scalable 1T MoS2 with low temperature-coefficient of resistance
Source: Sci Rep. 2018 Aug 20;8:12463. doi: 10.1038/s41598-018-30867-y (PMC6102259; doi:10.1038/s41598-018-30867-y)
Supplement: Supplementary file 1 — Supplementary Information [file 41598_2018_30867_MOESM1_ESM.pdf]

Supplementary Information

Stable and scalable 1T MoS_2 with low temperature-coefficient of resistance

Chithra H. Sharma, Ananthu P. Surendran, Abin Varghese and Madhu Thalakulam*

School of Physics, Indian Institute of Science Education & Research Thiruvananthapuram
Kerala, India 695551
* madhu@iisertvm.ac.in

S1: Table summarising other reported works on 1T-MoS_2

Sl. No.	Reference	Process	Stability	Electrical Measurements	Scalability
1.	Kim *et al.* (Ref 11)	Li intercalation	Not Available; Chemical process known to be unstable	300 K to 100 K TCR ~ 10^{-2} K^{-1}	1T domains among other phases
2.	Acerce *et al.* (Ref 19)	Li intercalation	5000 CV cycles. Chemical process known to be unstable	Highly conducting electrodes	1T Nanosheets
3.	Kapera *et al.* (Ref 25)	Li intercalation	Not Available Chemical process known to be unstable	300 K No gate response	1T for contacts
4.	Kang *et al.* (Ref 33)	Plasmon excitation in Au nano spheres	Not Available	Not Available	Not applicable for device fabrication.
5.	Zhu *et al.* (Ref 34)	Ar Plasma	1 week	Has gate dependence	1T: 2H ratio 40%, nm size domains
6.	Lin *et al.* (Ref 12)	Electron beam at high temperature	Not Available	Not Available	Small area, not scalable
7.	Katagiri *et al.* (Ref 32)	Ebeam irradiation	1 month	Schottky 2H-1T junction	Not scalable

S2: HR-TEM image of the same region showing back conversion from 1T to 2H during imaging

Before

After

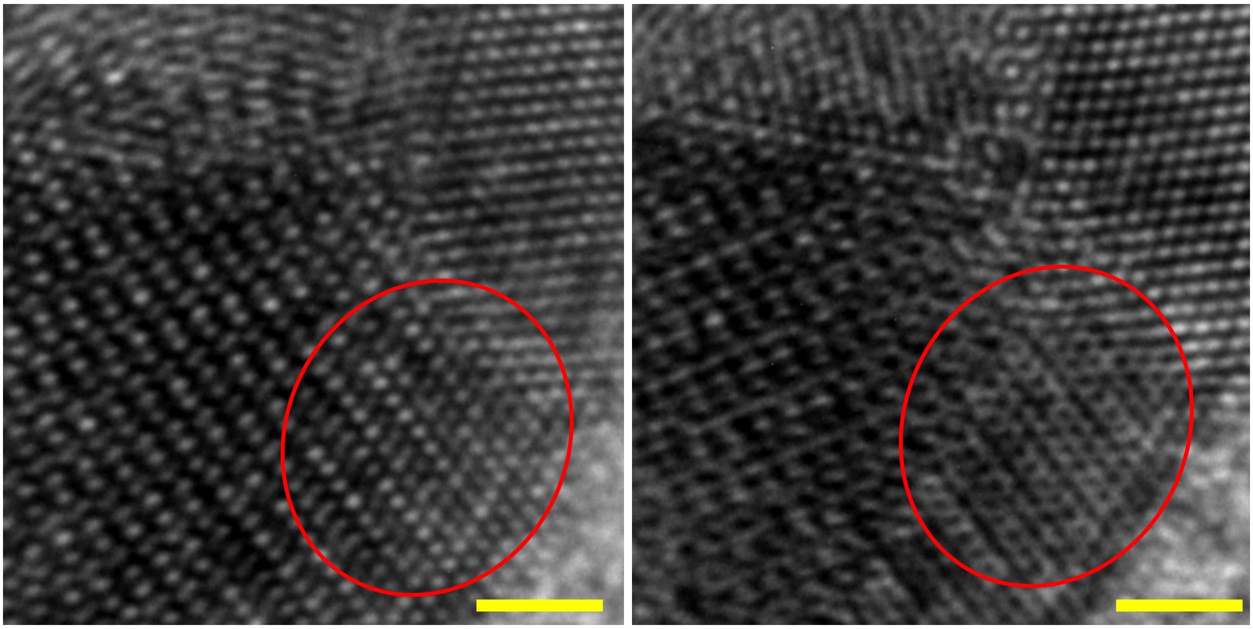

1T to 2H conversion can be seen in the region enclosed in red. Scale bar 2 nm

S3: HR-TEM showing 2H and 1T regions

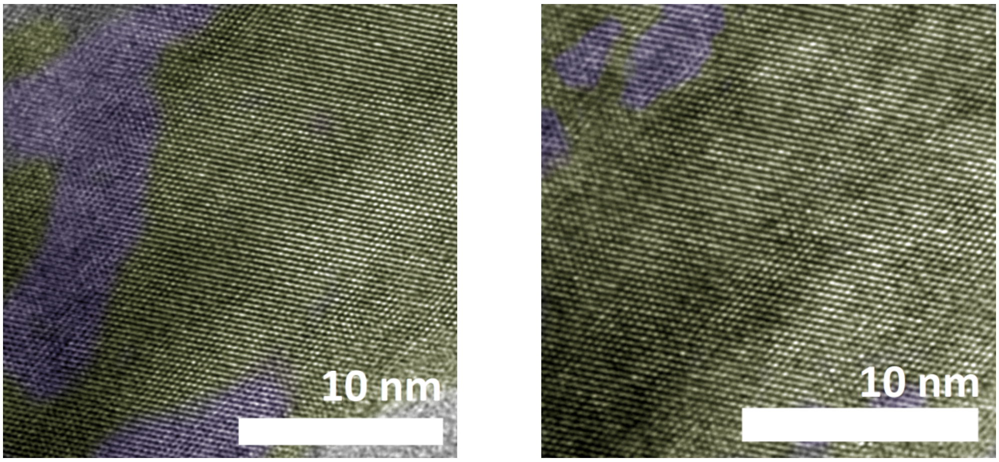

Representative HR-TEM images of plasma treated samples showing 1T (green) and 2H (purple) phases.

S4: Line profile showing Mo-Mo distance

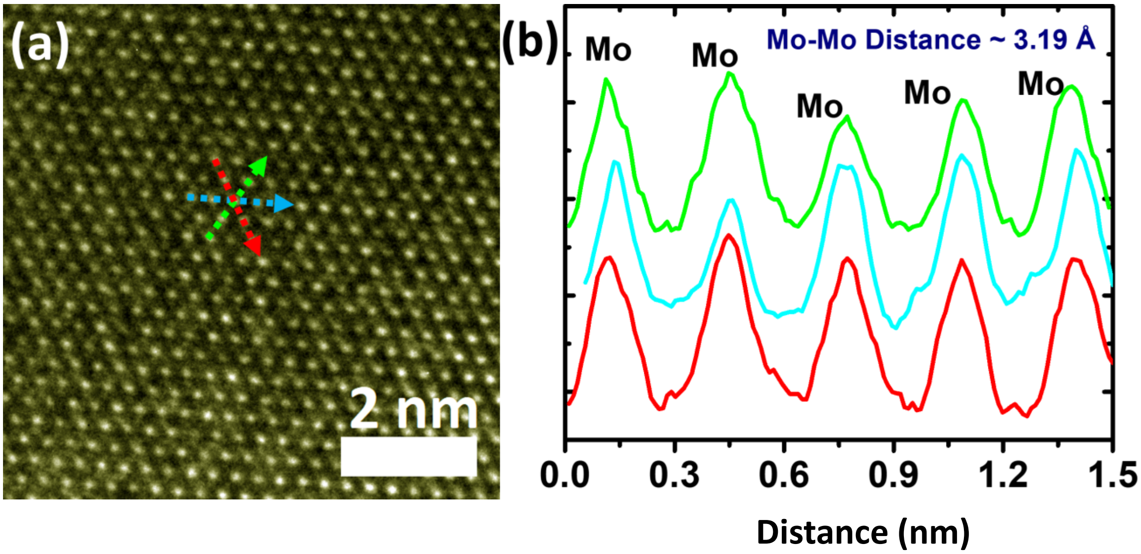

The Mo-Mo bond distances in three different directions showing identical values indicating that it is not the 1T' phase.

S5: Raman showing J1, J2 peaks post-plasma treatment

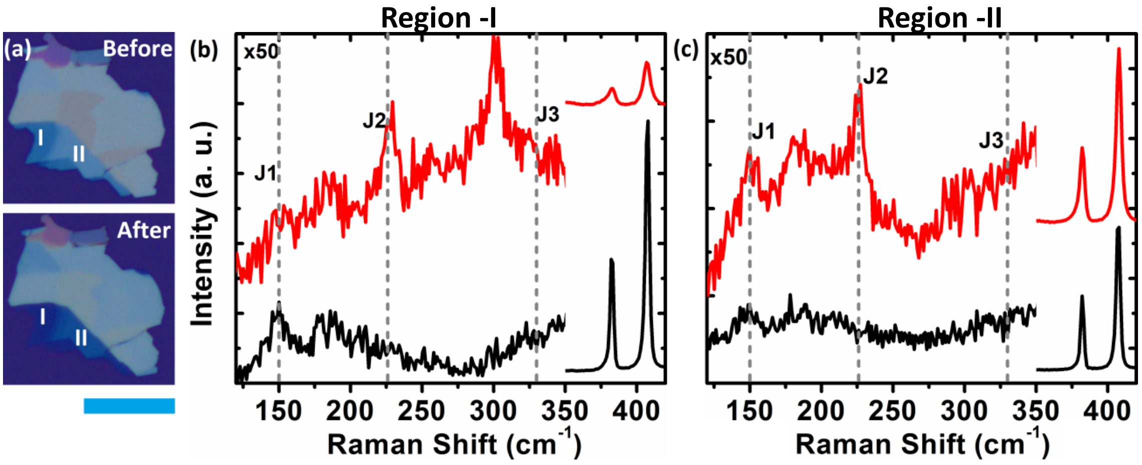

Raman from region I and II before (black) and after (red) plasma treatment show clear peaks corresponding to the J_1 and J_2 vibrational modes corresponding to the 1T phase . The region I post plasma corresponds to a monolayer.

S6: Raman spectra from the 1T region of sample in Fig. 4 (a)

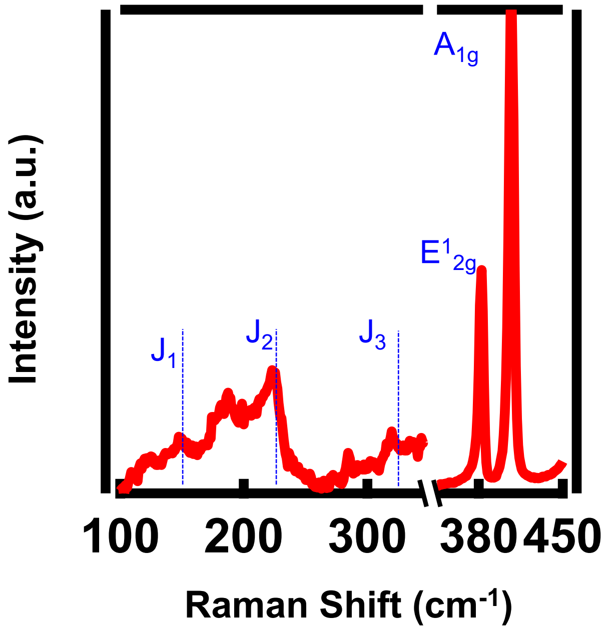

The Raman spectra from the 1T region of the sample in Fig. 4 (a) showing J_1, J_2, J_3, E^1_{2g} and A_{1g} peak positions

S7: Lithographically defined 1T and 2H regions

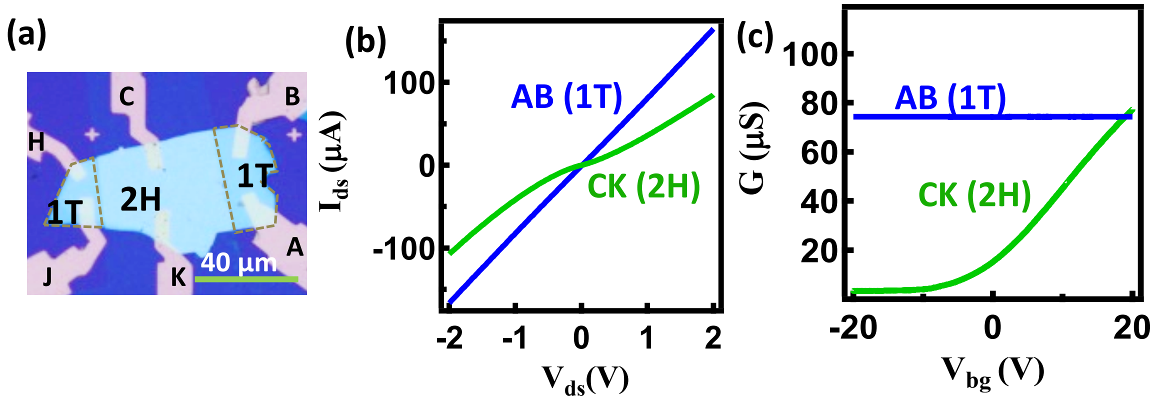

(a) Image of the sample with lithographically defined 2H and 1T regions. The 1T regions are marked with dashed lines. (b) I-V at 4 K for the 2H and 1T regions. The 1T region shows linear IV even at 4K whereas the 2H region shows Schottky behaviour. (c) The Conductance v/s back gate voltage for 1T and 2H regions at 4 K; 1T region shows no gate voltage dependence while the 2H shows variation with V_{bg} exhibiting n-type conduction.

S8: Gate response and IV from a plasma treated sample

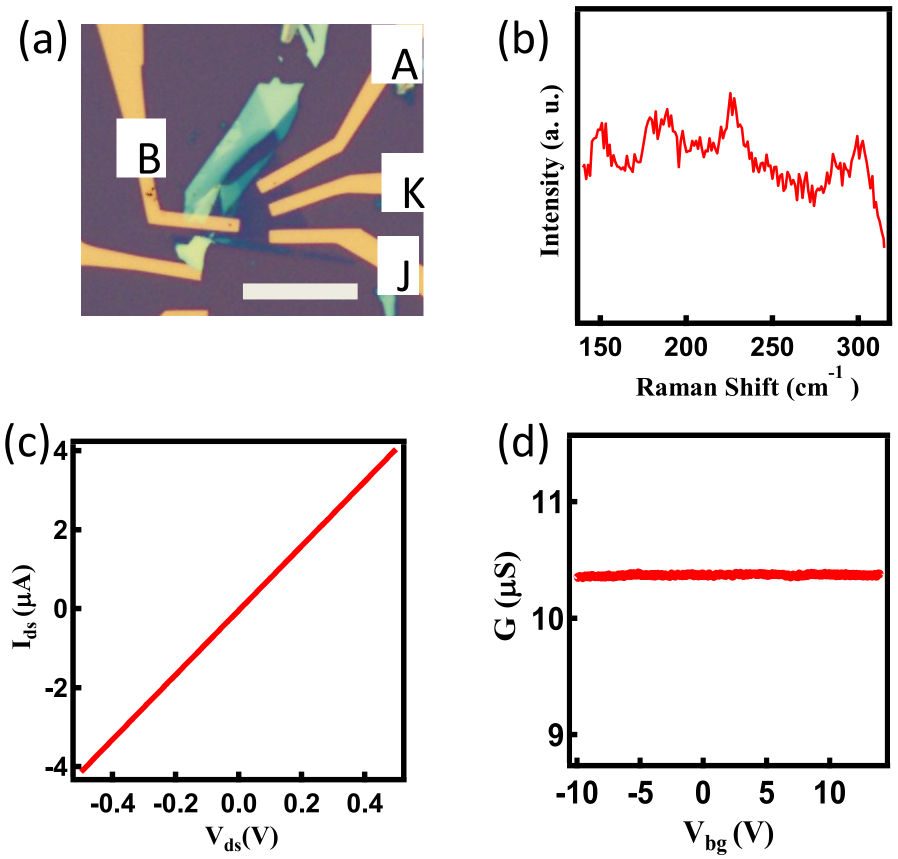

(a) Image of the 1T-MoS2 device prepared by plasma treatment. (b) Raman spectra showing clear peaks corresponding to the J_1 and J_2 vibrational modes corresponding to the 1T phase. (c) I-V characteristics at 300 K (d) Conductance vs back-gate voltage.

S9: Resistance at various time of the sample in Fig. 4(d)

Sl No	No. of days after sample fabrication	Resistance (Ω)
1	0	~ 70
2	22	70.76
3	58	70.1
4	92	~ 70

The time includes sample in the measurement chamber and the time sample was in ambience. Cumulatively the sample was exposed to ambience for ~ 1 month

S10: Raman post-plasma treatment without H_2

Ar alone

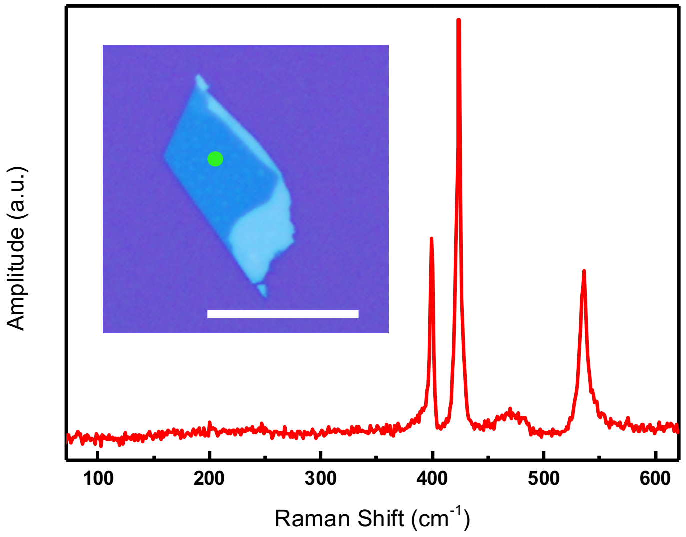

N_2 alone

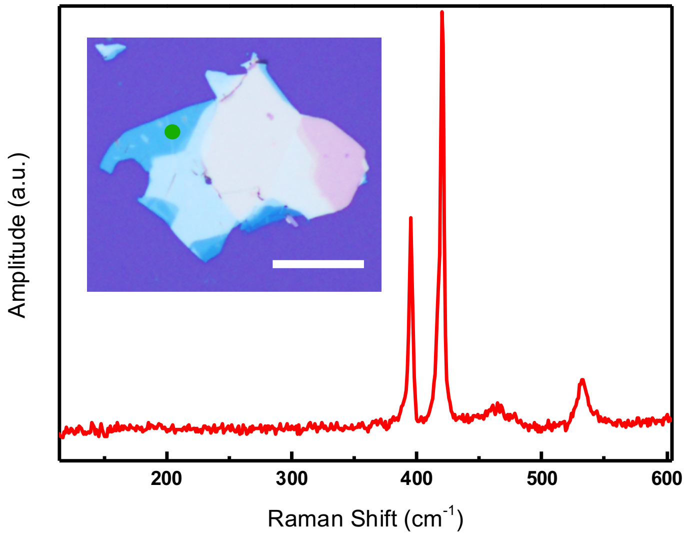

Raman spectra after exposing to Ar plasma and N_2 plasma showing no signatures of the J_1, J_2 or J_3 peaks. The inset shows post treatment image of the sample where the green spot shows the position from which the Raman was taken. Scale bar: 20 µm
